# Truck and Multivehicle Truck Accidents with Injuries Near Colorado Oil and Gas Operations

**DOI:** 10.3390/ijerph15091861

**Published:** 2018-08-28

**Authors:** Benjamin D. Blair, John Hughes, William B. Allshouse, Lisa M. McKenzie, John L. Adgate

**Affiliations:** 1 Colorado School of Public Health, University of Colorado, Aurora, CO 80045, USA; benjamin.blair@ucdenver.edu (B.D.B.); william.allshouse@ucdenver.edu (W.B.A.); lisa.mckenzie@ucdenver.edu (L.M.M.); 2 Independent Researcher, Frostburg, MD 21532, USA; jphughesjr@gmail.com

**Keywords:** oil and gas operations, trucking, transport, accidents, hydraulic fracturing

## Abstract

Unconventional and conventional oil and gas (O&G) operations raise public health concerns, such as the potential impacts from trucking activity in communities that host these operations. In this work, we used two approaches to evaluate accidents in relation to O&G activities in the State of Colorado. First, we calculated the rate of truck accidents by computing the ratio of accident count and county population. When comparing counties with increased O&G operations to counties with less activity, we found that counties with more activity have greater rates of truck traffic accidents per capita (Rate Ratio = 1.07, *p* < 0.05, 95% CI: 1.01–1.13). Second, we laid a grid over the eleven counties of interest and counted, for each cell, the number of truck accidents, the number of multivehicle accidents with injuries, the number of homes, and the number of O&G wells. We then applied hurdle count models, using the accident counts as the outcomes and the number of homes and number of wells as independent variables. We found that both independent variables are significant predictors of truck accidents and multivehicle truck accidents. These accidents are of concern since they can have an impact on the people who live near O&G operations.

## 1. Introduction

Oil and gas (O&G) operations have significantly increased in the United States over the past decade. These operations are raising public health concerns [1,2,3], as they often occur near or within residential areas [4]. Estimates of the rate of traffic accidents resulting from the transportation of personnel and materials to and from these sites is limited, and there is little data on the severity of these truck accidents.

The transport of materials related to O&G operations has been raised as a central area of concern among citizens residing near these operations [5]. Trucking activity around O&G operation sites is substantial due to the transport of large volumes of water, sand, equipment, and chemicals that are used in the process and the transport of the product from the site to refineries or pipelines [6,7,8,9]. To illustrate the substantial transport activities from O&G operations, it has been estimated that between 4315 and 6590 truck trips are required over the lifetime of a six-well pad [7]. The high density of wells, such as the 21,044 active wells in 2012 in the rapidly growing Denver Julesburg Basin, also influences the overall trucking activity in populated areas [4].

Increased trucking from O&G operations have resulted in potential illness and documented injuries and fatalities [1,10,11]. Previous research reported that a 10% increase in the rate of traffic accidents was found for every 10 new wells drilled in Pennsylvania [12]. In the Eagle Ford basin in Texas, an area with substantial O&G operations, traffic accidents increased 26% from 2009 to 2013 and fatalities and severe injuries increased 49% from 2009 to 2013 [13]. Motor vehicle accidents were found to be the leading cause of death of O&G industry workers in the United States and accounted for 28% of all O&G extraction work-related fatalities from 2003 to 2009 [11]. In Colorado, USA, a taskforce involving a wide range of O&G stakeholders reported “uniform agreement that one of the most serious impacts of O&G activity involves the use of large trucks and trailers” [14].

Data specific to O&G operations are sparse and methodological advances are needed to understand the spatial relationship between accidents from transport related to these operations. The goal of this study was to evaluate the relationship between truck accident incidence and O&G development in Colorado using two techniques. First, we evaluated the county-level incidence of truck accidents on a per capita basis from 2005 to 2013 and we compared the top 10 counties by oil production to the other counties in Colorado. Second, we applied a grid analysis to 11 counties of interest to evaluate the relationship between truck accidents, O&G operations, and homes [4]. We also included multivehicle truck accidents with an injury in the grid analysis to evaluate the potential harm to residents living in proximity to O&G operations. Through the evaluation of these goals, we seek to advance the understanding of the public health impacts from O&G trucking activity and offer the first evaluation of truck accidents in relation to O&G development within the State of Colorado. We also discuss potential prevention options pertinent to O&G operations.

## 2. Materials and Methods

### 2.1. County-Level Analysis

Data on truck accidents from 2005 to 2013 in Colorado were obtained using a Colorado Open Records Act request from the Colorado Department of Transportation. Available information included county, date, and location; number of injuries, fatalities, and vehicles involved; and date of any reported injuries. County populations were estimated using a linear regression of 2000, 2010, and 2015 U.S. Census Bureau data.

We selected the top 10 oil producing counties (Adams, Arapahoe, Cheyenne, Garfield, Larimer, Lincoln, Moffat, Rio Blanco, Washington, and Weld) in 2013 to compare to the remaining 54 counties in Colorado that have fewer O&G operations based on oil production [15]. Data from the Colorado Oil and Gas Conservation Commission (COGCC) Reports Portal indicates that 97.8% of oil production occurred in those 10 counties during 2013. Additional trucking is often needed for oil compared to natural gas production because gas pipeline infrastructure is typically more common than oil pipeline infrastructure [16].

The incidence of truck accidents per capita was found by dividing the annual number of accidents by the annual estimated county population. Other commonly used metrics, such as daily truck miles traveled, were not used as this would incorporate the increase in trucking activity from O&G operations. Population was used to account for the potential increase in trucking activity from other sources in the county. The rate ratio (RR) compared the incidence of truck accidents per population in counties with oil and gas activity to the counties without activity using the epitools package in R [17,18].

### 2.2. Grid Analysis

The data used for the grid analysis included truck accident data from the county-level analysis, well activity and location, and the location of homes in 11 counties of interest. Housing data was available based on a population analysis near O&G operations for Adams, Arapahoe, Boulder, Broomfield, Garfield, La Plata, Larimer, Logan, Mesa, Morgan, and Weld counties [4]. Home addresses based on 2012 were purchased from DataQuick and the latitude and longitude of each home address were found using Google API [4]. Homes with rooftop accuracy were included [4]. The count of homes is used as a proxy for traffic and population density independent of O&G operation activities. Well locations and counts were based on data from the COGCC that includes all wells with any listed activity at any time between 2005 and 2013 [19].

Grids of various sizes were overlaid on Colorado to increase the spatial resolution beyond that of the county-level data. Four grid sizes were chosen: 0.1 degrees by 0.1 degrees, 0.05 degrees by 0.05 degrees, 0.025 degrees by 0.025 degrees, and 0.01 degrees by 0.01 degrees, using modified R code [20]. The number of total truck accidents, multivehicle accidents including a truck and injury, homes, and O&G wells were counted for each grid cell.

A hurdle model was applied because (1) there were no truck accidents (i.e., outcomes) in a large proportion of the grids resulting in an outcome value of zero, and (2) the zero outcomes have exactly one source (a zero indicates that there were no trucks accidents) [21,22]. The hurdle model employed a logistic regression model for the incidence process, and a zero-truncated negative binomial regression model (with log link) for the prevalence process. We used the pscl package in R [23]. The incidence model is used to show whether more homes and/or wells are associated with a higher probability of truck accidents, or accidents with multiple vehicles with an injury, while the prevalence model shows if more homes and/or wells are associated with a change in number of truck accidents or accidents with multiple vehicles with an injury. The negative binomial model was necessary since the sample variance was much larger than sample mean. For the two finer resolution grids (0.01 by 0.01 degrees and 0.025 by 0.025 degrees), a very high percentage of cells with no truck accidents (zero values) led to computational difficulties and implausible results as the estimates of the overdispersion parameter, theta, indicate considerable overdispersion, making a Poisson model for prevalence counts inappropriate. We fit the models using the method of maximum likelihood. Both component models included an intercept term as well as main effects for homes and wells. Including higher order polynomial terms for homes and/or wells modestly improved model fit, but led to implausible and/or uninterpretable results.

## 3. Results

### 3.1. County-Level Analysis

**Figure 1:**
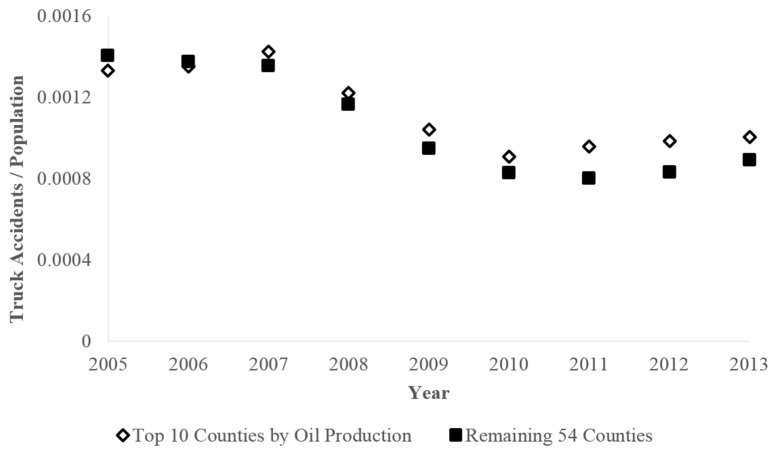
Rate of truck accidents per county population from 2005 to 2013 for the top 10 oil producing counties and the remaining 54 Colorado counties.

Our first analysis compared the incidence of truck accidents in Colorado’s top 10 producing oil counties to other Colorado counties. Over the nine-year period shown in Figure 1, the incidence of truck accidents per capita was higher in the top 10 oil producing counties compared to the remaining 54 counties in Colorado (Rate Ratio = 1.07, *p* < 0.05, 95% CI: 1.01–1.13). The drop in the rate ratio over time is likely driven by population increase in the top oil producing counties, while the relative increase in truck accidents appears to be related to the increase in unconventional oil and gas development between 2005 and 2016 [4,24,25].

### 3.2. Grid Analysis

For the 0.1 by 0.1 and the 0.05 by 0.05 grid we used 604 cells and 2267 cells, respectively, to cover the area of analysis. The count of the variables within a cell of interest are zero inflated, as shown by the summary information (Table 1). As the cell size decreases, such as with the 0.05 by 0.05 cells, the median value is zero for the variables of interest, which highlights the strongly skewed distribution. For the 0.05 by 0.05 degree and 0.1 by 0.1 degree grids, the counts of both homes and wells are significant predictors of the incidence and prevalence for total truck accidents using the hurdle model (Table 2). The incidence model shows that more homes and/or wells are associated with a higher probability of truck accidents while the prevalence model shows that more homes and/or wells are associated with a larger number of truck accidents.

Similar to the county level analysis, the cell count of both homes and wells are significant predictors of incidence and prevalence of multivehicle truck accidents with injuries for the 0.05 by 0.05 degree and 0.1 by 0.1 degree grids (Table 2). The incidence model shows that more homes and/or wells are associated with a higher probability of multivehicle truck accidents with an injury while the prevalence model shows that more homes and/or wells are associated with a larger number of multivehicle accidents with an injury.

## 4. Discussion

The increase in trucking from O&G operations into a densely populated area can impact the number of trucking accidents, thereby increasing risk to those living and working in these communities. In this work, we examined a period when oil and gas drilling was rapidly increasing, and our results suggest increased truck accidents related to the O&G activity in Colorado. We found that the top 10 oil producing counties in Colorado had higher truck accident rates than the remaining 54 counties. The county level rates were alike between the two groups of counties from 2005 to 2008. However, the difference in rates between the top producing oil counties and counties with less O&G operations increased from 2009 to 2013, when substantial O&G operation growth in Colorado occurred. To illustrate this relationship, Colorado had 28,952 conventional and unconventional active wells in 2005, 39,944 active wells in 2008, 37,311 active wells in 2009 and 50,067 active wells in 2013, for an overall increase of 21,115 active wells during this period [2,15]. While approximately 55% of the wells in Colorado are unconventional, nearly all the wells developed since the mid-2000s are unconventional and have used horizontal drilling and high-volume hydraulic fracturing.

From the grid analysis, we found that the count of number of homes and wells in an area are significant predictors of total truck accidents and multivehicle truck accidents with an injury. Through the use of active well counts as a proxy for trucking activity near O&G operations, we found that the O&G operation activity predicts trucking accidents and multivehicle trucking accidents with injuries after adjusting for the number of homes as proxy for population density. The results for multivehicle truck accidents with an injury could imply that the accidents are of greater severity and potentially greater risk to the general population near these O&G operations.

Truck transport is “considered the least safe mode of transporting oil” and options exist to reduce the use of trucks for petroleum transport, and the corresponding accidents from the transport of these materials [26]. The use of permanent pipelines to transport oil and waste and temporary pipelines to transport water to well pads may reduce truck traffic and mitigate some risk from trucking accidents [14]. For example, the USGAO (2014) found 14 fatalities from 2007 to 2011 involving pipeline incidents, while there were 3675 fatalities from trucking and 730 fatalities from railway transport of hazardous materials, including oil and gas [9]. These results indicate that pipelines may reduce the risks from the transportation of hazardous liquids. However, pipelines are often a politically contentious issue and carry risks. Railway transport may offer a feasible alternative, yet the catastrophic accidents from railway transport also raise concern [26]. Recent efforts are also moving toward tankless sites, which would reduce trucking traffic dedicated to liquids transport [27]. Other traffic accident prevention suggestions specific to O&G operations include avoiding transport during school zone hours and the use of alternative routes to circumvent schools, bus routes, and bike or walking paths [28].

The primary strength of this work is the ability to apply a geospatial analysis to statewide data from the Colorado Department of Transportation, the Colorado Oil and Gas information System (COGIS), census population information, and home locations. We were able to generate both county-level and grid level analyses. This work also advances the understanding of truck accident risk from O&G operations by outlining a geospatial and statistical approach to further understand the proximity of accidents to O&G operations. A limitation of this work is the lack of a direct relationship between the truck accidents recorded in Colorado and O&G activity. While fatal trucking accidents have been documented from O&G operations, the linking of any individual truck accident to O&G activities is impossible with the data used in this study [11]. Improved data and analyses would include trucking accidents directly related to O&G operations. Additionally, further temporal considerations may be needed, such as periods in the boom and bust cycle in particular, given the trends involving the O&G industry. The count of homes from 2012 may not be indicative of all homes and traffic patterns and future research could introduce changes in construction patterns to the analysis. Well activity may have also varied greatly, ranging from small low producing well sites to large sites that could potentially have significant pad preparation, vertical and horizontal drilling, hydraulic fracturing, and other production activities.

Future research on this topic could establish a more direct link between O&G operations and trucking accidents. These future research efforts could be accomplished by requiring drivers working directly for O&G operations to report an accident to an agency, such as a state agency tasked with overseeing O&G operations, to record these incidents. Future studies could also collect and code the written narratives (such as from police reports) to document specific incidents related to O&G operations. Lastly, we also recommend that future investigations explore evaluating driver characteristics, such as fatigue, driving unknown routes, inexperienced drivers, etc., that may increase trucking accidents around O&G operations. To meet these future research objectives, we recommend areas with significant O&G operations to consider additional reporting of the occurrence and severity of transportation accidents as well.

## 5. Conclusions

We reported on the county-level rate of trucking accidents and their association with a rapidly increasing population and expanding O&G operations. Accidents from the trucking related to O&G operations are documented sources of injuries and fatalities and this offers further insight into the accident rate around these operations in Colorado. We found that a refined spatial analysis through the use of grids provides insight into not only how the O&G activity can influence the prevalence or incidence of trucking accidents at a state or regional level, but this approach could also further evaluate the direct risk of these activities to the general population in close proximity to O&G development. Due to the increased risk of truck accidents near O&G operations, systematic reporting and tracking of these incidents will be important for developing and evaluating accident prevention strategies. Other transportation modes, such as pipelines and rail transport, should be considered as approaches to minimize the risk from traffic accidents.

## Figures and Tables

**Table 1:** Summary of results for counts of homes, wells, truck accidents, and multivehicle truck accidents with an injury for the 0.1 by 0.1 and 0.05 by 0.05 grids.

Cell Size	Variable	Minimum	Median	Mean	Maximum
0.1 by 0.1	Homes	0	7	1132	45,164
	O&G Wells	0	2	66	1372
	Truck Accidents	0	0	24	1848
	Multivehicle Truck Accident with Injury	0	0	4	314
0.05 by 0.05	Homes	0	0	299	18,610
	O&G Wells	0	0	18	494
	Truck Accidents	0	0	6	802
	Multivehicle Truck Accident with Injury	0	0	1	162

**Table 2:** On left, truck accidents predicted by hurdle model for 0.05 by 0.05 grid and 0.1 by 0.1 grid. On right, truck accidents involving multiple vehicles and an injury predicted by hurdle model for 0.05 by 0.05 grid and 0.1 by 0.1 grid.

	Truck Accidents				Truck Accidents Involving Multiple Vehicles and an Injury			
	0.05 by 0.05 degree grid ^1^		0.1 by 0.1 degree grid ^2^		0.05 by 0.05 degree grid ^3^		0.1 by 0.1 degree Grid ^4^	
Prevalence model coefficients (truncated negbin with log link):								
	Estimate	Std. Error	Estimate	Std. Error	Estimate	Std. Error	Estimate	Std. Error
Intercept	1.61 ***	0.14	2.46 ***	0.14	0.27	0.26	1.28 ***	0.16
Homes	35.57 ***	3.56	26.38 ***	4.01	23.64 ***	3.07	16.94 ***	2.39
Wells	10.35 ***	2.64	8.00 ***	2.04	9.76 **	3.28	8.18 ***	1.86
Log(theta)	−1.39 ***	0.19	−1.17 ***	0.21	−1.33 ***	0.34	−0.58 *	0.25
Incidence model coefficients (negbin with logit link):								
Intercept	−0.67 ***	0.08	0.97 ***	0.27	−1.44 ***	0.078	0.06	0.20
Homes	148.76 ***	16.74	125.72 ***	27.66	147.00 ***	14.62	118.8 ***	21.2
Wells	20.13 ***	2.38	10.45 ***	3.12	18.54 ***	2.29	8.85 ***	2.52
Theta: count (95% CI)	0.25 (0.17–0.37)		0.31 (0.21–0.47)		0.26 (0.14–0.52)		0.56 (0.34–0.92)	

*** indicates *p* < 0.001, ** indicates *p* < 0.01, * indicates *p* < 0.05. ^1^ For the 0.05 by 0.05 grid, the value of the log likelihood at convergence was −3240.82. The corresponding intercept-only model had log likelihood equal to −3557. The AIC values were 6495.64 and 7121, respectively, which yielded a model probability <0.001. ^2^ For the 0.1 by 0.1 grid, the value of the log likelihood at convergence was −1549.31. The corresponding intercept-only model had log likelihood equal to −1675.60. The AIC values were 3112.62 and 3357.21, respectively, which yielded a model probability <0.001. ^3^ For the 0.05 by 0.05 grid, the value of the log likelihood at convergence was −1754.16. The corresponding intercept-only model had log likelihood equal to −2049.26. The AIC values were 3522.32 and 4104.52, respectively, which yielded a model probability <0.001. ^4^ For the 0.1 by 0.1 grid, the value of the log likelihood at convergence was −895.09. The corresponding intercept-only model had log likelihood equal to −1025.10. The AIC values were 1804.17 and 2056.20, respectively, which yielded a model probability <0.001.

## References

[1] Adgate J.L., Goldstein B.D., McKenzie L.M. (2014). Potential Public Health Hazards, Exposures and Health Effects from Unconventional Natural Gas Development. Environ. Sci. Technol..

[2] Blair B.D., McKenzie L.M., Allshouse W.B., Adgate J.L. (2017). Is Reporting “Significant Damage” Transparent? Assessing Fire and Explosion Risk at Oil and Gas Operations in the United States. Energy Res. Soc. Sci..

[3] Werner A.K., Vink S., Watt K., Jagals P. (2015). Environmental Health Impacts of Unconventional Natural Gas Development: A Review of the Current Strength of Evidence. Sci. Total Environ..

[4] McKenzie L.M., Allshouse W.B., Burke T., Blair B.D., Adgate J.L. (2016). Population Size, Growth, and Environmental Justice near Oil and Gas Wells in Colorado. Environ. Sci. Technol..

[5] Ellis C., Theodori G.L., Petrzelka P., Jackson-smith D., Luloff A.E. (2016). Unconventional Risks: The Experience of Acute Energy Development in the Eagle Ford Shale. Energy Res. Soc. Sci..

[6] Ewen C., Borchardt D., Richter S., Hammerbacher R. (2012). Hydrofracking Risk Assessment. https://www.ufz.de/export/data/2/201588_Abschlussbericht%20Ex_HydrofrackingRiskAssessment_120611.pdf.

[7] Goodman P.S., Galatioto F., Thorpe N., Namdeo A.K., Davies R.J., Bird R.N. (2016). Investigating the Traffic-Related Environmental Impacts of Hydraulic-Fracturing (Fracking) Operations. Environ. Int..

[8] Patterson L.A., Maloney K.O. (2016). Transport of Hydraulic Fracturing Waste from Pennsylvania Wells: A County-Level Analysis of Road Use and Associated Road Repair Costs. J. Environ. Manag..

[9] USGAO (2014). Oil and Gas Transportation: Department of Transportation is Taking Actions to Address Rail Safety, but Additional Actions Are Needed to Improve Pipeline Safety. http://www.gao.gov/assets/670/665991.txt.

[10] McCawley M.A. (2017). Does Increased Traffic Flow around Unconventional Resource Development Activities Represent the Major Respiratory Hazard to Neighboring Communities? Knowns and Unknowns. Curr. Opin. Pulm. Med..

[11] Retzer K.D., Hill R.D., Pratt S.G. (2013). Motor Vehicle Fatalities among Oil and Gas Extraction Workers. Accid. Anal. Prev..

[12] Graham J., Irving J., Tang X., Sellers S., Crisp J., Horwitz D., Muehlenbachs L., Krupnick A., Carey D. (2015). Increased Traffic Accident Rates Associated with Shale Gas Drilling in Pennsylvania. Accid. Anal. Prev..

[13] Rahm D., Fields B., Farmer J. (2015). Transportation Impacts of Fracking in the Eagle Ford Shale Development in Rural South Texas: Perceptions of Local Government Officials. J. Rural Community Dev..

[14] (2015). Colorado Oil and Gas Task Force, Final Report. https://www.colorado.gov/pacific/sites/default/files/atoms/files/2015OilGasTaskForceReport.pdf.

[15] COGCC Reports Portal. http://cogcc.state.co.us/COGCCReports/production.aspx?id=MonthlyOilProdByCounty.

[16] Rahm D. (2011). Regulating Hydraulic Fracturing in Shale Gas Plays: The Case of Texas. Energy Policy.

[17] Tomas A., Aragon J., Fay M.P., Wollschlaeger D., Omidpanah A. (2017). Package “epitools”. https://cran.r-project.org/web/packages/epitools/index.html.

[18] Ihaka R., Gentleman R. (1996). R: A Language for Data Analysis and Graphics. J. Comput. Graph. Stat..

[19] Colorado Oil and Gas Information System (COGIS). https://cogcc.state.co.us/cogis/.

[20] Aggregating Points to Grid Using R. https://gis.stackexchange.com/questions/48416/aggregating-points-to-grid-using-r.

[21] Welsh A., Cunningham R., Donnelly C., Lindenmayer D. (1996). Modelling the Abundance of Rare Species: Statistical Models for Counts with Extra Zeros. Ecol. Model..

[22] Khemka G., Roberts S., Higgins T. (2017). The Impact of Changes to the Unemployment Rate on Australian Disability Income Insurance Claim Incidence. Risks.

[23] Zeileis A., Kleiber K., Jackman S. (2008). Regression Models for Count Data in R. J. Stat. Softw..

[24] U.S. Energy Information Administration (2018). Hydraulically Fractured Horizontal Wells Account for Most New Oil and Natural Gas Wells. https://www.eia.gov/todayinenergy/detail.php?id=34732.

[25] National Oil and Gas Gateway. http://www.noggateway.org/explore.

[26] Abkowitz M. (2016). The Impact of Fracking on Freight. http://www.wistrans.org/cfire/documents/CFIRE_0915_FinalReport.pdf.

[27] Anadarko Sustainable Development. http://www.anadarko.com/Responsibility/Sustainable-Development/.

[28] Witter R., McKenzie L., Towle M., Stinson K., Scott K., Newman L., Adgate J. (2010). Health Impact Assessment for Battlement Mesa, Garfield County Colorado. https://www.garfield-county.com/public-health/documents/1%20%20%20Complete%20HIA%20without%20Appendix%20D.pdf.

